# Efficacy of Crizotinib, Ceritinib, and Alectinib in ALK-Positive Non-Small Cell Lung Cancer Treatment: A Meta-Analysis of Clinical Trials

**DOI:** 10.3390/cancers12030526

**Published:** 2020-02-25

**Authors:** Tung Hoang, Seung-Kwon Myung, Thu Thi Pham, Boyoung Park

**Affiliations:** 1 Department of Cancer Biomedical Science, National Cancer Center Graduate School of Cancer Science and Policy, Goyang 10408, Korea; 75256@ncc.re.kr; 2 Division of Cancer Epidemiology and Management, National Cancer Center Research Institute, Goyang 10408, Korea; 3 Department of Family Medicine and Center for Cancer Prevention and Detection, National Cancer Center Hospital, Goyang 10408, Korea; 4 Health Data Science Program, Institute of Public Health, Charité Universitätsmedizin Berlin, 10117 Berlin, Germany; thuphamhup@gmail.com; 5 Molecular Epidemiology Research Group, Max Delbrück Center for Molecular Medicine (MDC), 13092 Berlin, Germany; 6 Department of Medicine, Hanyang University College of Medicine, Seoul 04763, Korea; hayejine@hanmail.net

**Keywords:** ALK inhibitors, non-small cell lung cancer, crizotinib, ceritinib, alectinib

## Abstract

This study aimed to evaluate the efficacy of anaplastic lymphoma kinase (ALK)-inhibitors in the treatment of ALK-positive non-small cell lung cancer (NSCLC) by using a meta-analysis of clinical trials. We searched PubMed, EMBASE, Cochrane Library, and Clinicaltrials.gov by using keywords related to the topic in August 2018. The pooled effect sizes were calculated based on a random-effects model. We also performed subgroup meta-analysis by types of ALK inhibitors (crizotinib, ceritinib, and alectinib). A total of 20 clinical trials with 10 single-arm trials and 10 double-arm trials were included in the final meta-analysis. The median overall survival (OS), progression-free survival (PFS), overall response rate (ORR), disease control rate (DCR), 1 year survival rate, and 2 year survival rate were 19.14 months, 8.47 months, 62%, 78%, 74%, and 62%, respectively. ALK inhibitors showed a significantly superior efficacy compared with chemotherapy (hazard ratio (HR) for OS, 0.83; HR for PFS, 0.43; rate difference (RD) for ORR, 0.23; and RD for DCR, 0.10). The current meta-analysis of clinical trials showed the significant efficacy of ALK inhibitors in the treatment of ALK-positive NSCLC. Further head-to-head trials are needed to compare their efficacy with other types of NSCLC treatment regimens. PROSPERO registration: CRD42018085987.

## 1. Introduction

Non-small cell lung cancer (NSCLC) accounts for approximately 85–90% of lung cancers, which are the most common fatal malignancy and leading cause of cancer mortality worldwide [1,2]. It is reported that the median overall survival (OS) with platinum-based chemotherapy is approximately 7.5–28.2 months among advanced NSCLC patients, and the median progression-free survival (PFS) is approximately 2.1–6.9 months [3]. In the last decade, the treatment of advanced NSCLC has shifted into determining molecular subtypes of the disease based on oncogenic drivers, which has led to the introduction of several newly approved biological agents [4].

One of them is crizotinib, initially designed for a mesenchymal–epithelial transition factor (MET) inhibitor in 2007, which prompted the development of anaplastic lymphoma kinase (ALK) target therapy [5]. It became the first ALK inhibitor to be approved by the Food and Drug Administration (FDA) in 2011 for standard first-line therapy in ALK-positive NSCLC, which accounts for approximately 2–7% of patients diagnosed with NSCLC [6]. However, the progression of brain metastases and resistance were the biggest challenges during crizotinib treatment [7]. In 2014 and 2015, next-generation ALK inhibitors such as ceritinib and alectinib were approved by the FDA for the treatment of ALK-positive NSCLC patients who have developed or are intolerant to crizotinib [8,9].

Since the initial development of ALK inhibitors, subsequent clinical trials on the efficacy of the ALK inhibitors have been published [10,11,12,13,14,15,16,17,18,19,20,21,22,23,24,25,26,27,28,29]. Several systematic reviews and meta-analyses have also been reported [30,31,32,33]. However, Fan J et al. mainly investigated the efficacy and safety of alectinib, although they reported the findings of ORR and DCR for alectinhib in the ALK inhibitor-naïve or crizotinib-resistant patients [31]. The OS, which shows primary outcomes for clinical trials of oncology as well as other efficacy outcomes such as 1-year survival rate and 2 year survival rate, has not been investigated [31]. Although a network meta-analysis of the same research group, focusing on the comparative treatment effect of ALK inhibitors, reported the aggregated estimates for some outcomes, it included phase I or phase I/II studies that reported responses affected by dose differences [30]. Two publications reported the results from a qualitative review and a quantitative meta-analysis mainly based on the small number of four or five individual studies, respectively, from a search of only PubMed [32,33].

The current study aimed to investigate the efficacy of ALK inhibitors in patients with ALK-positive NSCLC using a meta-analysis of clinical trials.

## 2. Results

### 2.1. Selection of Relevant Studies

**Figure 1:**
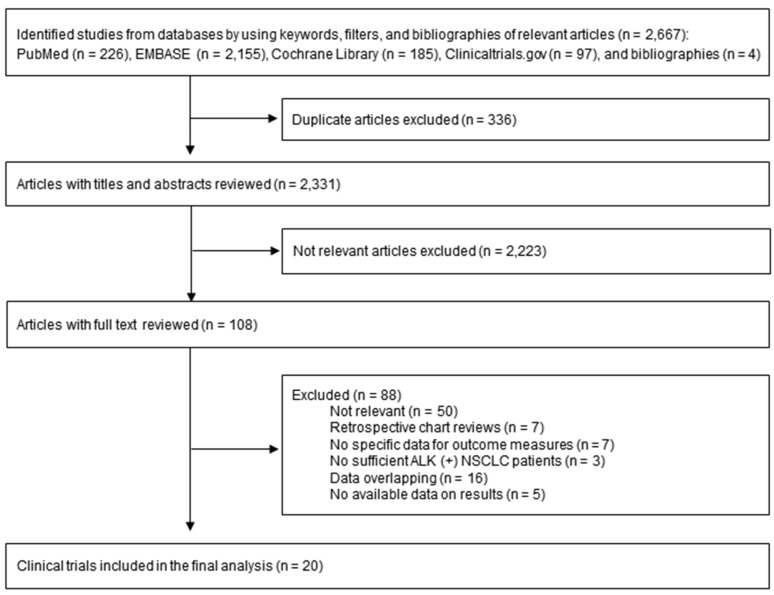
Flow diagram for selection of relevant clinical trials.

By the initial search of four databases (Pubmed, Embase, Cochrane Library, and Clinicaltrials.gov) and hand-searching relevant bibliographies, we identified 2667 articles (Figure 1). After excluding 336 duplicated articles, two authors independently reviewed and excluded 2223 articles that did not satisfy the selection criteria based on each article’s title and abstract. Among them, 88 articles were excluded after reviewing the full text of the remaining 108 articles. The reasons for exclusion were not relevant (*n* = 50), retrospective chart reviews (*n* = 7), no specific data for outcome measures (*n* = 7), no sufficient ALK-positive NSCLC (*n* = 3), data overlapping (*n* = 16), and no available data on results (*n* = 5). A total of 20 clinical trials were included in the final analysis with 18 studies [10,11,12,13,14,15,16,17,18,19,20,21,22,23,24,26,28,29] in English and two studies [25,27] in Chinese.

### 2.2. General Characteristics of Studies

The general characteristics of the included studies are shown in Table 1. Except for 13 global multicenter trials [10,11,14,16,17,18,19,20,21,22,23,24,29], the seven remaining studies were conducted in China [12,25,26,27] and Japan [13,15,28]. Four studies [10,12,21,26] (1344 patients), three studies [11,16,28] (406 patients), and three studies [14,15,23] (243 patients) used a single arm design for the efficacy of crizotinib, ceritinib, and alectinib, respectively. Five studies [18,19,20,25,27] (967 patients), two studies [22,24] (607 patients), one study [29] (72 patients), and two studies [13,17] (510 patients) investigated the efficacy of crizotinib versus chemotherapy, ceritinib versus chemotherapy, alectinib versus chemotherapy, and alectinib versus crizotinib, respectively.

### 2.3. Risk of Bias for Randomized, Double-Blind, Placebo-Controlled Trials

Table 2 shows the assessment of the risk of bias for randomized, double-blind, placebo-controlled trials. Most of the trials demonstrated a low risk of bias in less than five out of seven items, except for one trial with low risk of bias in six items [18].

**Figure 2:**
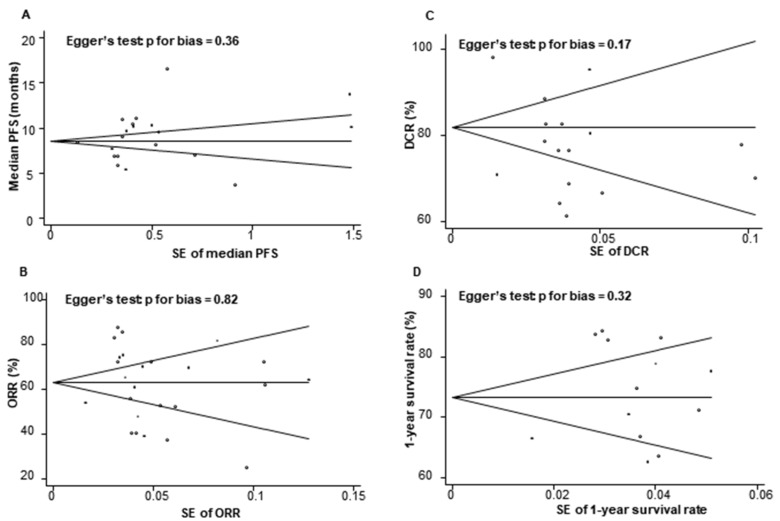
Begg’s funnel plots and Egger’s test for publication bias by different outcomes. (**A**): PFS, progression-free survival, (**B**) ORR, overall response rate, (**C**) DCR, disease control rate, (**D**) 1-year survival rate; SE, standard error.

Begg’s funnel plot and Egger’s test showed no evidence for publication bias (*p* > 0.05 for PFS, overall response rate (ORR), disease control rate (DCR), and 1 year survival rate; Figure 2).

### 2.4. Efficacy of ALK Inhibitors in Patients with ALK-Positive NSCLC by Type of Outcomes and Type of ALK Inhibitors

Table 3 shows the efficacy of ALK inhibitors in patients with ALK-positive NSCLC in the subgroup meta-analysis type of ALK inhibitors for each outcome in single-arm or double-arm trials. Overall, ceritinib showed shorter OS and PFS and lower ORR and DCR, compared with crizotinib and alectinib.

**Figure 3:**
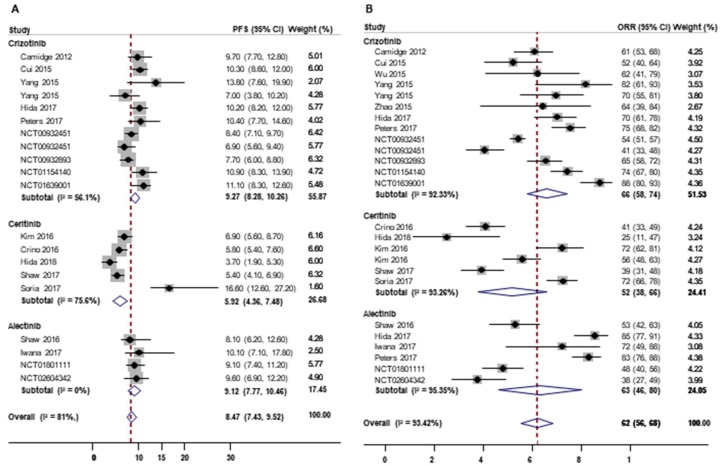
Efficacy of ALK inhibitors in treatment of ALK-positive non-small cell lung cancer (NSCLC) by type of outcome and type of ALK inhibitors. (**A**) PFS, progression-free survival (months), (**B**) ORR, overall response rate (%).

In the meta-analysis of all the included studies, the median OS was 19.14 months (95% confidence interval (CI), 16.42–21.85; I^2^ = 51%; *n* = 5), and the median PFS was 8.47 months (95% CI, 7.43–9.52; I^2^ = 80%; *n* = 20; Figure 3A). The pooled ORR, DCR, 1-year survival rate, and 2-year survival rates were 62% (95% CI, 56–68; I^2^ = 93%; *n* = 25; Figure 3B), 78% (95% CI, 71–84; I^2^ = 95%; *n* = 16), 74% (95% CI, 70–79; I^2^ = 82%; *n* = 13), and 62% (95% CI, 49–76; *n* = 3), respectively.

### 2.5. Efficacy of ALK Inhibitors Compared with Chemotherapy in Patients with ALK-Positive NSCLC by Type of Outcomes and Type of ALK Inhibitors

Shown in Table 4, ALK inhibitors showed superior efficacy in the treatment of ALK-positive NSCLC compared with chemotherapy in OS (hazard ratio (HR), 0.83; 95% CI, 0.72–0.97; I^2^ = 0%; *n* = 5), PFS (HR, 0.43; 95% CI, 0.35–0.54; I^2^ = 65%; *n* = 6), ORR (rate difference (RD), 23%; 95% CI, 17–29, I^2^ = 53%; *n* = 8), and DCR (RD, 10%; 95% CI, 4–16, I^2^ = 45%; *n* = 6).

In the subgroup meta-analysis by type of ALK inhibitors, similar findings were observed in PFS (HR, 0.45; 95% CI, 0.38–0.54; *n* = 3 for crizotinib vs. chemotherapy; HR, 0.52; 95% CI, 0.43–0.64; *n* = 2 for ceritinib vs. chemotherapy; and HR, 0.15; 95% CI, 0.08–0.29; *n* = 1 for alectinib vs. chemotherapy), ORR (RD, 19%; 95% CI, 12–26; *n* = 5 for crizotinib vs. chemotherapy; RD, 28%; 95% CI, 16–40; *n* = 2 for ceritinib vs. chemotherapy; and RD, 29%; 95% CI, 18–40; *n* = 1 for alectinib vs. chemotherapy), and DCR (RD, 6%; 95% CI, 1–11; *n* = 4 for crizotinib vs. chemotherapy and RD, 18%; 95% CI, 8–28; *n* = 1 for ceritinib vs. chemotherapy). However, crizotinib and ceritinib did not significantly improve the OS (HR, 0.83; 95% CI, 0.69–1.00, *n* = 3 and HR, 0.85; 95% CI, 0.62–1.16, *n* = 2, respectively). Also, crizotinib and ceritinib showed no significant efficacy in 1 year survival rate and 2 year survival rate, respectively.

**Figure 4:**
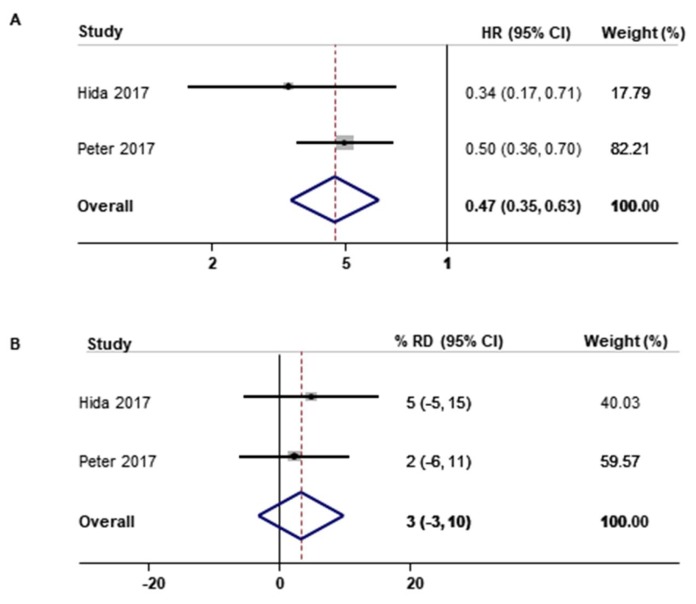
Efficacy of alectinib versus crizotinib in treatment of ALK-positive NSCLC by type of outcome. (**A**) progression-free survival, (**B**) overall survival rate; HR, hazard ratio, RD, rate difference, 95% CI, 95% confidence interval.

Further, the pooled risk of disease progression in two studies was significantly lower in patients treated with alectinib than those treated with crizotinib (HR for PFS, 0.47; 95% CI, 0.35–0.63; I^2^ = 0%; Figure 4A). Meanwhile, there was no difference in the efficacy of alectinib versus crizotinib in ORR (Figure 4B).

## 3. Discussion

### 3.1. Summary of Findings

In the current meta-analysis of clinical trials, we demonstrated that the median OS, PFS, ORR, DCR, 1 year survival rate, and 2 year survival rate for ALK inhibitors including crizotinib, ceritinib, and alectinib in the treatment of ALK-positive NSCLC was 19.14 months, 8.47 months, 62%, 78%, 74%, and 62%, respectively. In the subgroup analysis by type of ALK inhibitor, overall ceritinib showed shorter OS and PFS and lower ORR and DCR compared with crizotinib and alectinib. As compared with chemotherapy, ALK inhibitors showed superior efficacy in the treatment of ALK-positive NSCLC. 

### 3.2. Comparison with Previous Studies

Our findings are consistent with those from retrospective chart review studies. El. Din et al. reported that crizotinib showed a 1 year survival rate of 71.2% and an objective response rate of 70.9% [34]. Bendaly et al. reported that the ORR for ceritinib was 69% and median PFS was 12.9 months [6]. In a large, multi-country medical chart review (*n* = 1471) with seven countries, there was a significant improvement in complete response (odds ratio (OR), 2.65; 95% CI, 1.69–4.15) and a significant reduction in recurrence/progression (OR, 0.38; 95% CI, 0.24–0.59) [35].

Recently, a network meta-analysis reported higher response (ORR, 64%; 95% CI, 59-69 and DCR, 85%; 82–88) and PFS (9.2 months; 95% CI, 8.18–10.22 months) than those in our study [30]. In comparison with chemotherapy, ALK inhibitors showed a significantly longer PFS with the pooled HR (95% CIs) of 0.71 (0.66–0.76) for crizotinib, 0.75 (0.69–0.83) for ceritinib, and 0.50 (0.43–0.58) for alectinib [30]. Especially, alectinib was found to decrease the risk of ALK-positive NSCLC progression (HR, 0.70; 95% CI, 0.61–0.80) compared with crizotinib in the network meta-analysis [30].

However, in the recent meta-analysis of five randomized trials, ALK-targeted therapy performed better in PFS (HR = 0.48; 95% CI, 0.42–0.55), but not for OS (HR, 0.88, 95% CI, 0.72–1.07) [32], while another meta-analysis of four trials reported superior therapeutic outcomes regarding the increased 1 year and 2 year OS, PFS, and ORR, compared to chemotherapy [33].

### 3.3. Possible Mechanisms

There are several mechanisms regarding the therapeutic efficacy of ALK inhibitors in the treatment of ALK-positive NSCLC. In ALK-positive NSCLC, ALK-echinoderm microtubule-associated protein-like 4 (EML4) fusion protein activates the RAS/mitogen-activated protein kinase (MAPK), phosphatidylinositol 3-kinase (PI3K)/AKT, and janus kinase (JAK)/signal transducer, and the activator of transcription 3 (STAT) pathways are reported to play the important role in the development of NSCLC [5,36]. In the meantime, it has been shown that ALK inhibitors can bind the ALK protein to prevent the activation of NSCLC pathways [5,36].

Regarding the resistance to crizotinib, several biological mechanisms have been proposed [37]. Resistance may arise through the pathway of selective copy number gain or gene amplification (ALK-dependent) [37,38]. However, about 70% of crizotinib resistance is attributable to the abnormal activation of alternative signaling pathways involving ALK-independent growth, not to identifiable secondary resistance mutations or ALK copy number alterations [39,40]. Even though crizotinib significantly improved the treatment response compared with conventional chemotherapy, the disease progression in the central nervous system has still frequently occurred [41]. Thus, second-generation ALK inhibitors including ceritinib and alectinib with higher selectivity were designed to overcome resistance issues related to crizotinib and improve the activity of treatment therapy in the central nervous system [42].

### 3.4. Strengths and Limitations

To the best of our knowledge, this is the first meta-analysis to investigate the overall profile of ALK inhibitors’ efficacy in the treatment of ALK-positive NSCLC. We also estimated the efficacy of crizotinib, ceritinib, and alectinib compared with chemotherapy, respectively.

However, there are several limitations in the current study. First, although we found that ALK inhibitors improved PFS in considerable trials, OS was not sufficiently evaluated due to a relatively small number of trials. As a surrogate, PFS can be evaluated faster, with fewer patients. However, OS is still considered to be the gold standard in clinical trials of oncology drugs [43]. Second, substantial heterogeneity was observed in the meta-analysis of single-arm studies for all the outcomes and double-arm studies for PFS, ORR, and DCR outcomes (I^2^ > 50%). Last, due to a small number of trials, we were unable to conduct the head-to-head comparisons among different types of ALK inhibitors.

## 4. Materials and Methods

The protocol for this systematic review and meta-analysis was registered with the International Prospective Register of Systematic Reviews (PROSPERO registration number: CRD 42018085987).

### 4.1. Literature Search

We searched Pubmed, EMBASE, Cochrane library, and Clinicaltrials.gov databases from their inception until August 2018, limiting it to human subjects and clinical trials. The keywords for the literature search were as follows: ‘crizotinib’, ‘ceritinib’, ‘alectinib’, and ‘ALK inhibitor’ for intervention factors; ‘non-small cell lung cancer’ for outcome factor; ‘clinical trial’, ‘randomized controlled trial’ for study type. The bibliographies of relevant articles were also reviewed to identify additional studies. The format (abstract or full text) and language of publications were not restricted.

### 4.2. Study Selection and Eligibility Criteria

We included clinical trials that investigated the efficacy of three ALK inhibitors (crizotinib 250 mg bid, ceritinib 750 mg·qd, or alectinib 300/600 mg·bid) in ALK-positive NSCLC patients and reported findings on at least one of the following outcomes, i.e., OS (time from randomization to death), PFS (time from randomization to disease progression), ORR (complete response plus partial response), DCR (complete response plus partial response and stable disease), 1 year survival rate, and 2 year survival rate. The longer survival time in OS and PFS, or higher percentage of ORR, DCR, 1 year survival rate and 2 year survival rate a treatment has, the higher its efficacy. For studies using the same data, completely duplicated records were excluded, while partially duplicated records were combined to obtain the full information. Based on the eligibility criteria, two investigators (Hoang and Myung) independently selected studies to be included in the analysis.

### 4.3. Statistical Analyses

We used OS, PFS, ORR, DCR, 1 year survival rate, and 2 year survival rate with 95% CIs from individual studies to calculate the pooled effect time/ effect rate. For studies using the comparison group, we calculated a pooled HR for OS, PFS and a RD for ORR, DCR, 1 year survival rate, and 2 year survival rate between the two groups.

In order to measure heterogeneity across studies, we used Higgins I^2^, which estimates the percentage of total variation across studies. Negative values of I^2^ are set to zero; I^2^ ranges between 0% (no observed heterogeneity) and 100% (maximal heterogeneity) [39]. A random-effects model was used to calculate a pooled effect size [40].

Publication bias where 10 or more studies were available was examined by using Begg’s funnel plot and Egger’s test [41,44]. We also estimated the risk of bias for eligible studies based on the Cochrane Risk of Bias Tool [39]. We used the Stata SE version 14.0 software (StataCorp, College Station, Texas, USA) for the statistical analysis.

## 5. Conclusions

In conclusion, the current meta-analysis of clinical trials suggests the superior efficacy of ALK inhibitors including crizotinib, ceritinib, and alectinib in the treatment of ALK-positive NSCLC, compared with chemotherapy. Further randomized controlled trials are needed to evaluate the efficacy of different types of ALK inhibitors in head-to-head trials and the efficacy of those compared with other types of NSCLC treatment regimens.

## Figures and Tables

**Table 1:** General characteristics of clinical trials included in the final analysis.

Study	Enrollment Period	Regimen	No. pts	OS (95% CI) (Months)	PFS (95% CI) (Months)	ORR (%)	DCR (%)	1-Year Survival Rate	2-Year Survival Rate
**Single-arm study**									
Camidge 2012 [10] (PROFILE 1001)	08/2008–06/2011 (US, Australia, South Korea)	Crizotinib	143	-	9.7 (7.7–12.8)	60.8	82.5	74.8	-
Cui 2015 [12]	06/2013–10/2014 (China)	Crizotinib	67	-	10.3 (8.6–12.0)	52.2	64.2	77.6	-
Yang 2015 [26]	12/2010–08/2014 (China)	Crizotinib	22 46	-	13.8 (7.6–19.9) 7.0 (3.8–10.2)	81.8 69.6	-	-	65.0 50.0
Crino 2016 [11] (ASCEND-2)	12/2012–09/2013 (51 global sites)	Ceritinib	140	15.6 (13.6–24.2)	5.8 (5.4–7.6)	38.6	77.1	63.8	-
Kim 2016 [16] (ASCEND-1)	01/2011–07/2013 (11 countries)	Ceritinib	83 163	-	- 6.9 (5.6–8.7)	72 56	-	83 67	-
Shaw 2016 [23] (NCT01871805)	09/2013–08/2014 (US, Canada)	Alectinib	87	-	8.1 (6.2–12.6)	52.9	66.7	71	-
Iwana 2017 [15]	09/2014–12/2015 (Japan)	Alectinib	18	-	10.1 (7.1–17.8)	72.2	77.8	-	-
Hida 2018 [28] (ASCEND-9)	08/2015–03/2017 (Japan)	Ceritinib	20	-	3.7 (1.9–5.3)	25	-	-	-
NCT00932451 [21] (PROFILE 1005)	01/2010–03/2015 (21 countries)	Crizotinib	908 158	21.8 (19.4–24.0) 16.9 (13.4–21.5)	8.4 (7.1–9.7) 6.9 (5.6–9.4)	54.1 40.5	70.8 61.4	66.5 62.4	-
NCT01801111 [14]	06/2013–10/2014 (16 countries)	Alectinib	138	-	9.1 (7.4–11.2)	47.8	68.8	-	-
**Double-arm study**									
Wu 2015 [25]	06/2010–11/2014 (China)	Crizotinib vs. Pemetrexed/docetaxel/gemcitabine/paclitaxel + platinum	21 21	-	-	61.9 28.6	-	-	-
Zhao 2015 [27]	01/2012–12/2013 (China)	Crizotinib vs. Dexamethasone + docetaxel	14 14	-	-	64.3 21.4	-	-	-
Hida 2017 [13] (J-ALEX)	11/2014–08/2015 (Japan)	Alectinib vs. Crizotinib	103 104	-	- 10.2 (8.2–12.0)	85 70	98.1 88.5	-	-
Peters 2017 [17] (ALEX)	08/2014–01/2016 (98 global sites)	Alectinib vs. Crizotinib	152 151	-	- 10.4 (7.7–14.6)	82.9 75.5	-	84.3 82.5	-
Shaw 2017 [22] (ASCEND-5)	06/2013–11/2015 (20 countries)	Ceritinib vs. Pemetrexed/docetaxel	115 116	18.1 (13.4–23.9) 20.1 (11.9–25.1)	5.4 (4.1–6.9) 1.6 (1.4–2.8)	39.1 6.9	76.5 36.3	-	-
Soria 2017 [24] (ASCEND-4)	08/2013–05/2015 (28 countries)	Ceritinib vs. Cisplatin/carboplatin	189 187	-	16.6 (12.6–27.2) 8.1 (5.8–11.1)	72.5 26.7	-	-	70.6 58.2
NCT00932893 [18] (PROFILE 1007)	09/2009–03/2012 (22 countries)	Crizotinib vs. Pemetrexed/docetaxel	173 174	21.7 (18.9–30.5) 21.9 (16.8–26.0)	7.7 (6.0–8.8) 3.0 (2.6–4.3)	65.3 19.5	64.2 38.5	70.4 66.7	-
NCT01154140 [19] (PROFILE 1014)	01/2011–11/2013 (31 countries)	Crizotinib vs. Pemetrexed + cisplatin/carboplatin	172 171	-	10.9 (8.3–13.9) 7.0 (6.8–8.2)	74.4 45.0	78.5 68.4	83.5 78.4	-
NCT01639001 [20]	09/2012–06/2015 (5 Asia countries)	Crizotinib vs. Pemetrexed + cisplatin/carboplatin	104 103	-	11.1 (8.3–12.6) 6.8 (5.7–7.0)	87.5 45.6	82.7 73.8	79.3 79.5	-
NCT02604342 [29]	11/2015–01/2017 (15 countries)	Alectinib vs. Pemetrexed/docetaxel	72 35	- -	9.6 (6.9–12.2) 1.4 (1.3–1.6)	37.5 2.9	80.6 28.6	- -	- -

**Table 2:** Summary of risk of bias assessment for randomized, double-blind, placebo-controlled trials (*n* = 10).

Study	Random Sequence Generation	Allocation Concealment	Blinding of Participants and Personnel	Blinding of Outcome Assessment	Incomplete Outcome Data	Selective Reporting	Other Bias	No. of Low Risk of Bias
Wu 2015 [25]	Unclear risk	Unclear risk	Unclear risk	Unclear risk	Low risk	Unclear risk	Low risk	2
Zhao 2015 [27]	Unclear risk	Unclear risk	Unclear risk	Unclear risk	Low risk	Unclear risk	Low risk	2
Hida 2017 [13] (J-ALEX)	Low risk	Low risk	Unclear risk	Low risk	Unclear risk	Unclear risk	Low risk	4
Peters 2017 [17] (ALEX)	Low risk	Low risk	Unclear risk	Low risk	Unclear risk	Unclear risk	Low risk	4
Shaw 2017 [22] (ASCEND-5)	Low risk	Low risk	Unclear risk	Low risk	Unclear risk	Unclear risk	Low risk	4
Soria 2017 [24] (ASCEND-4)	Low risk	Low risk	Unclear risk	Low risk	Unclear risk	Unclear risk	Low risk	4
NCT00932893 [18] (PROFILE 1007)	Low risk	Low risk	Unclear risk	Low risk	Low risk	Low risk	Low risk	6
NCT01154140 [19] (PROFILE 1014)	Low risk	Low risk	Unclear risk	Low risk	Unclear risk	Unclear risk	Low risk	4
NCT01639001 [20]	Low risk	Low risk	Unclear risk	Low risk	Unclear risk	Unclear risk	Low risk	4
NCT02604342 [29]	Low risk	Low risk	Unclear risk	Low risk	Unclear risk	Unclear risk	Low risk	4

**Table 3:** Efficacy of ALK inhibitors in patients with ALK-positive non-small cell lung cancer by type of ALK inhibitors for each outcome.

Outcome	No. of Groups	Period/Rate (95% CI)	I^2^ (%)
**Time period (months)**			
OS [11,18,21,22]	5	19.14 (16.42–21.85)	50.5
Crizotinib [18,21]	3	20.22 (16.94–23.50)	54.3
Ceritinib [11,22]	2	16.86 (13.13–20.59)	0.0
**PFS (months)** [10,11,12,13,14,15,16,17,18,19,20,21,22,23,24,26,28,29]	20	8.47 (7.43–9.52)	80.1
Crizotinib [10,12,13,17,18,19,20,21,26]	11	9.27 (8.28–10.26)	56.1
Ceritinib [11,16,22,24,28]	5	5.92 (4.36–7.48)	75.6
Alectinib [14,15,23,29]	4	9.12 (7.77–10.46)	0.0
**Rate (%)**			
**ORR** [10,11,12,13,14,15,16,17,18,19,20,21,22,23,24,25,26,27,28,29]	25	62 (56–68)	93.4
Crizotinib [10,12,13,17,18,19,20,21,25,26,27]	13	66 (58–74)	92.2
Ceritinib [11,16,22,24,28]	6	52 (38–66)	93.3
Alectinib [13,14,15,17,23,29]	6	63 (46–80)	95.4
**DCR** [10,11,12,13,14,15,18,19,20,21,22,23,25,28,29] [10,11,12,13,14,15,18,19,20,21,22,23,25,28,29]	16	78 (71–84)	94.8
Crizotinib [10,12,13,18,19,20,21,25]	8	78 (71–85)	90.9
Ceritinib [11,22,28]	3	76 (71–81)	0.0
Alectinib [13,14,15,23,29]	5	79 (63–95)	95.4
**1-year survival rate** [10,11,12,16,17,18,19,20,21,23]	13	74 (70–79)	85.3
Crizotinib [10,12,17,18,19,20,21]	8	75 (69–81)	86.7
Ceritinib [11,16]	3	71 (60–83)	85.0
Alectinib [17,23]	2	81 (76–86)	0.0
**2-year survival rate** [24,26]	3	62 (49–76)	69.0
Crizotinib [26]	2	55 (43–66)	0.0
Ceritinib [24]	1	70 (64–76)	NA

OS, overall survival; PFS, progression-free survival; ORR, overall response rate; DCR, disease control rate; 95% CI, 95% confidence interval; NA, not applicable.

**Table 4:** Efficacy of ALK inhibitors compared with chemotherapy in patients with ALK-positive non-small cell lung cancer by type of ALK inhibitors for each outcome.

Outcome	No of Groups	Effect size (95% CI)	I^2^ (%)
**Effect size: Hazard ratio**			
OS [18,19,20,22,24]	5	0.83 (0.72–0.97)	0.0
Crizotinib [18,19,20]	3	0.83 (0.69–1.00)	0.0
Ceritinib [22,24]	2	0.85 (0.62–1.16)	19.1
**PFS** [18,19,20,22,24,29]	6	0.43 (0.35–0.54)	64.7
Crizotinib [18,19,20]	3	0.45 (0.38–0.54)	0.0
Ceritinib [22,24]	2	0.52 (0.43–0.64)	0.0
Alectinib [29]	1	0.15 (0.08–0.29)	NA
**Effect size: Rate difference (%)**			
**ORR** [18,19,20,22,24,25,27,29]	8	23 (17–29)	52.7
Crizotinib [18,19,20,25,27]	5	19 (12–26)	36.5
Ceritinib [22,24]	2	28 (16–40)	65.4
Alectinib [29]	1	29 (18–40)	NA
**DCR** [18,19,20,22,25,29]	6	10 (4–16)	44.8
Crizotinib [18,19,20,25]	4	6 (1–11)	0.0
Ceritinib [22]	1	18 (08–28)	NA
Alectinib [29]	1	18 (06–30)	NA
**1-year survival rate** [18,19,20]			
Crizotinib [18,19,20]	3	1 (−4, 6)	0.0
**2-year survival rate** [22]			
Ceritinib [22]	1	5 (−3, 13)	NA

OS, overall survival; PFS, progression-free survival; ORR, overall response rate; DCR, disease control rate; 95% CI, 95% confidence interval; NA, not applicable.
